# Herpes Simplex Virus Esophagitis in a Patient Receiving Long-Term Nasal Corticosteroids: A Rare Case

**DOI:** 10.7759/cureus.66631

**Published:** 2024-08-11

**Authors:** Georgios Vougiouklakis, Aris P Agouridis, Konstantinos Alexakis, Andreas Mamilos, Nikolaos Spernovasilis

**Affiliations:** 1 Department of Internal Medicine, German Medical Institute, Limassol, CYP; 2 School of Medicine, European University Cyprus, Nicosia, CYP; 3 Department of Histopathology, German Medical Institute, Limassol, CYP; 4 Department of Infectious Diseases, German Medical Institute, Limassol, CYP

**Keywords:** nonsystemic corticosteroids, corticosteroids, esophagitis, hsv, herpes simplex virus

## Abstract

Herpetic esophagitis (HE), primarily caused by the herpes simplex virus (HSV)-1, is most commonly encountered in immunocompromised hosts, although it has been occasionally observed in immunocompetent patients. In the immunocompromised setting, it is typically correlated with human immunodeficiency virus (HIV) infection, malignancy, chemotherapy and radiotherapy, solid organ transplant, as well as the use of systemic corticosteroids and other immunosuppressive agents. We present the case of a 35-year-old patient on hemodialysis due to diabetic nephropathy who, after having received intranasal corticosteroids for three weeks, developed nausea, vomiting, and epigastric pain. Gastroscopy and subsequent biopsy revealed ulcerative esophagitis compatible with herpetic infection. Immunohistochemistry was negative for cytomegalovirus (CMV), while subsequent quantitative polymerase chain reaction (PCR) testing was positive for HSV-1, establishing the diagnosis of HSV esophagitis. After a 14-day course of valacyclovir, complete relief of symptoms was achieved. Herpetic esophagitis may occur in immunocompetent persons, whereas intranasal corticosteroids cannot be ruled out as potential contributors. Symptoms such as odynophagia, dysphagia, and fever in that setting warrant further investigation.

## Introduction

Herpetic esophagitis (HE) constitutes the second most common cause of esophagus infection, following *Candida* esophagitis. It is typically caused by herpes simplex virus-1 (HSV-1), although HSV-2 esophagitis has also been reported [1].

Usually manifested in the setting of immune suppression, especially impaired cellular immunity, HE has been mainly correlated with human immune deficiency virus (HIV) infection, hematologic and solid tumor malignancies, as well as other immunocompromising conditions [2,3]. However, cases in immunocompetent individuals are increasingly reported [4]. No case associating herpetic esophagitis with intranasal corticosteroids has been previously published [3,5]. We herein present a rare case of a patient with well-regulated chronic health issues, without overt cellular immunity compromise, who developed herpetic esophagitis after a long course of intranasal corticosteroids.

## Case presentation

**Figure 1:**
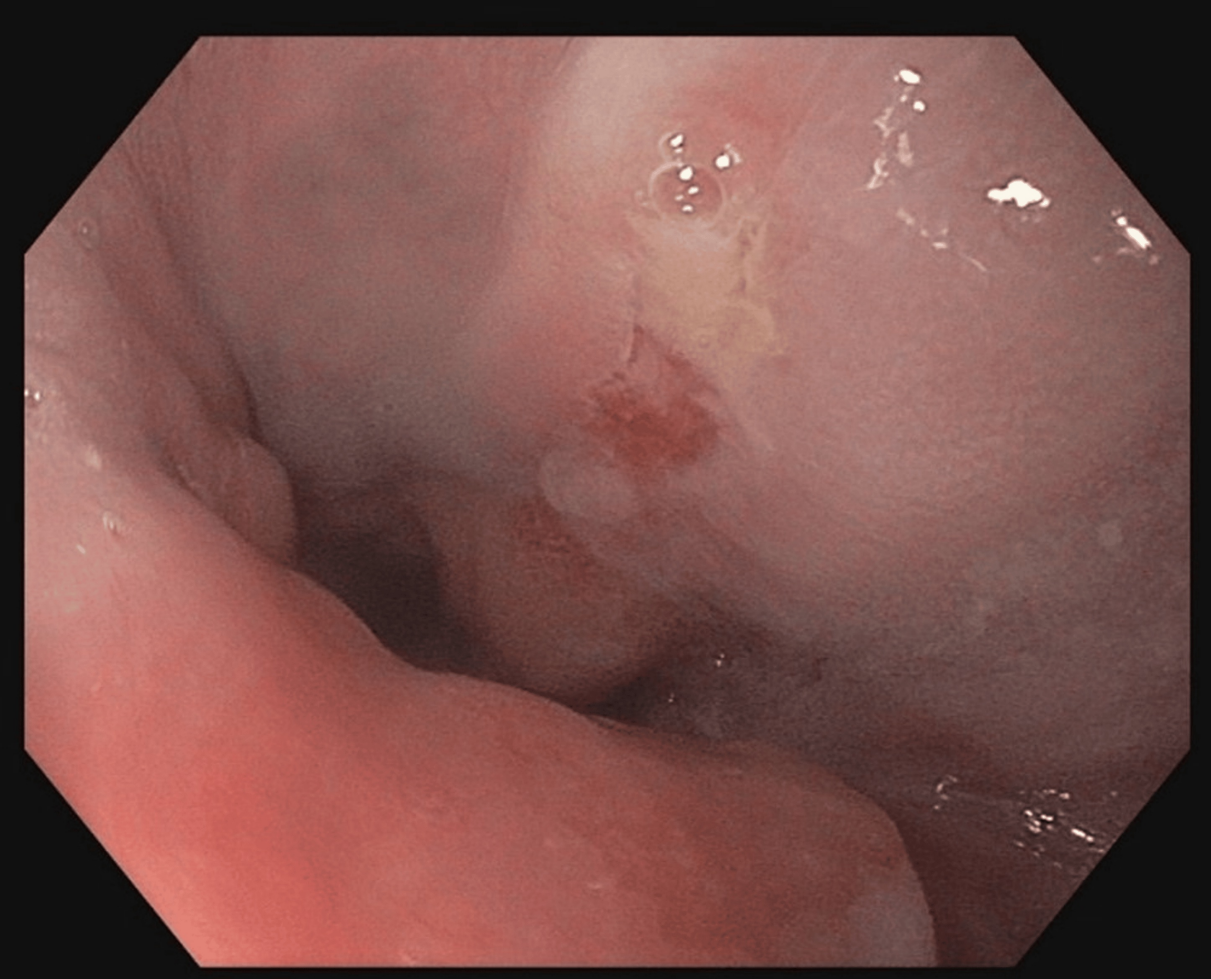
Endoscopy of the lower part of the esophagus. Endoscopy revealed a solitary ulcer, partially covered with exudate, just above the gastroesophageal junction.

A 35-year-old male patient with a medical history of thyroidectomy due to Grave’s disease, dyslipidemia, recurrent allergic rhinitis, and type I diabetes mellitus since the age of five with subsequent end-stage renal disease (ESRD) on hemodialysis for the past six years, presented with intermittent retrosternal and epigastric pain, nausea and vomiting during the previous month. His recent history was notable for prolonged episodes of allergic rhinitis over a period of two months before the onset of symptoms. During the second month, the patient was treated with nasal mometasone twice daily for three weeks until approximately a week before the onset of the aforementioned symptoms. The physical examination was unremarkable, apart from mild epigastric tenderness. The laboratory investigation was also unremarkable. Additionally, diabetes seemed to be adequately regulated (HbA1c:7.1%) three months before presentation. Esophagogastroduodenoscopy (EGD) revealed a solitary ulceration covered with exudate at the distal part of the esophagus, along with second-grade esophagitis and mild antrum gastritis (Figure 1). 

**Figure 2:**
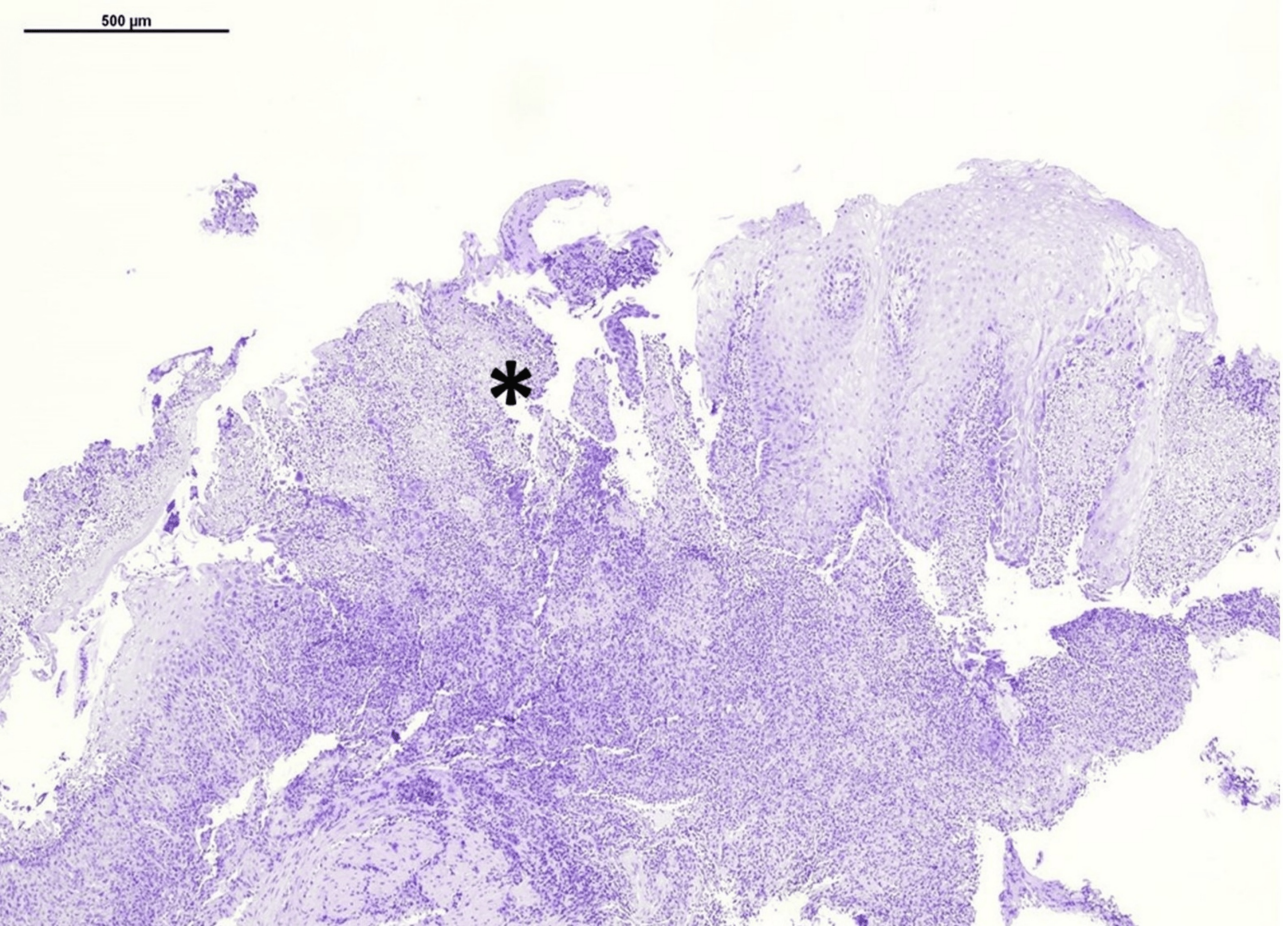
Esophagus histopathology. Ulcerative inflammation (*) of the esophagus. H.E. stain, 40x.

**Figure 3:**
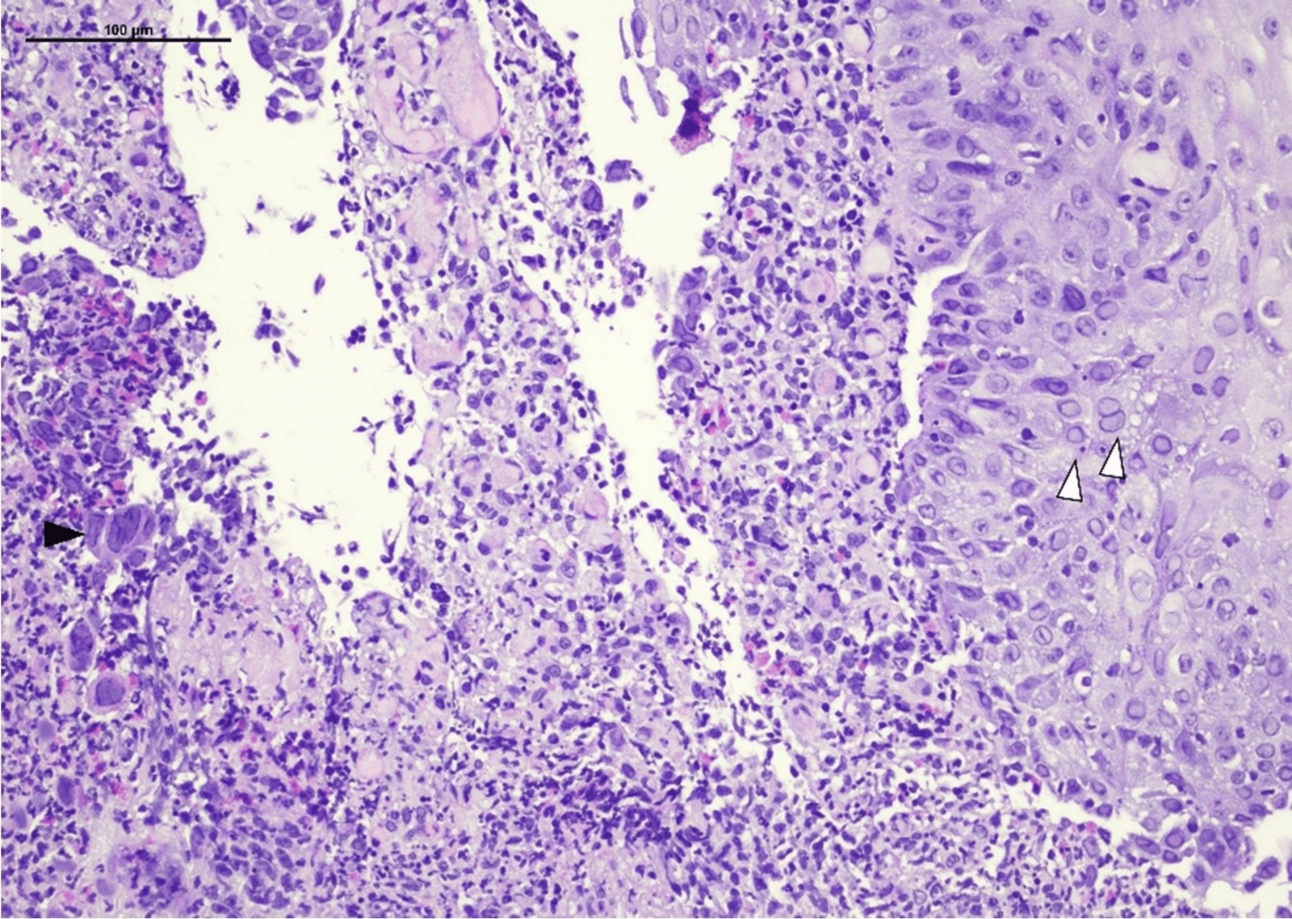
Esophagus histopathology. Ulcerative esophagitis with multinucleated cells (black arrowhead) and nuclear inclusions (white arrowheads). H.E. stain, 200x.

Histology showed duodenitis with a single ulcer and mild gastritis without* Helicobacter pylori* presence, as well as ulcerative esophagitis with neutrophil infiltration of the esophageal mucosa, giant multinucleated epithelial cells and eosinophilic intranuclear inclusions consistent with HSV infection (Figures 2, 3).

Immunohistochemistry was negative for cytomegalovirus (CMV) infection, while polymerase chain reaction (PCR) was positive for HSV, confirming the diagnosis of HE. Therefore, treatment with valacyclovir 500 mg qD for 14 days ensued without complications, leading to gradual and complete remission of symptoms. Follow-up with clinical examination and interview monthly for the first three months after completion of treatment and every two months subsequently for a total duration of one year was unremarkable. Due to clinical improvement and the patient's wishes, a subsequent gastroscopy was not performed.

## Discussion

Infectious esophagitis is the third most common cause of inflammation of the esophagus, following gastroesophageal reflux disease (GERD) and eosinophilic esophagitis (EoE). HSV is the second most frequently reported pathogen, following *Candida* species and trailed by CMV. Less commonly, infectious esophagitis has been attributed to HIV, varicella zoster virus (VZV), human papillomavirus (HPV), tuberculosis, and rarely to other bacteria such as gram-negative bacilli and Enterobacterales, *Staphylococcus* and *Streptococcus* species, as well as *Aspergillus* and *Histoplasma* species [6].

HSV-1, associated with oropharyngeal infection, causes herpetic esophagitis in most cases, while HSV-2, associated with urogenital infection, causes it less frequently [7]. The incidence is 1.8% in immunosuppressed individuals, and under the age of 40, a male predominance (3:1) has been reported [1,2]. Occurrence typically concerns the immunosuppressed state, after viral reactivation on the grounds of latent infection. The most prevalent risk factors include HIV infection, hematologic and solid tumor malignancies, bone marrow or solid organ transplantation, systematic inflammatory diseases, cancer chemotherapy, radiotherapy, long-term systemic corticosteroids, and immunosuppressive agents. Cases of patients associating chronic kidney disease (CKD), hemodialysis, and non-systemic use of corticosteroids with herpetic esophagitis, are extremely rare [1,3,6]. In the immunocompetent, it is increasingly reported as a self-limiting primary infection, occurring from local spread following oropharyngeal, urogenital, or skin lesions, in persons having esophageal mucosal injury and possibly with the contribution of a stressor that transiently compromises immunity [4,8]. Predisposing factors include GERD, the presence of foreign bodies, alcoholism, burns, malnourishment, and EoE [6,9]. Association with allergic rhinitis has also been implied in one case [10].

The most typical clinical manifestations of herpetic esophagitis include acute odynophagia, dysphagia, epigastric or retrosternal pain, and fever, as well as nausea and vomiting. Fever, cough, and sore throat may precede these symptoms. Other manifestations, such as malaise, anorexia, stomatitis, concomitant skin lesions, and bleeding, have also been reported [5,8,11]. Diagnosis is mainly based on endoscopy, which reveals elevated plaques with or without exudate, erosions, and ulcerations, usually in the mid to distal esophagus and subsequent biopsy. Histology findings, as observed in our patient, include the presence of large multinucleated epithelial cells (ballooning), macrophage sequestration, and characteristic eosinophilic inclusion bodies consisting of nucleic acid and protein material (Cowdry A inclusion bodies). Serology, immunohistochemistry, and tissue PCR may augment diagnosis [6]. 

Regarding the immunocompetent, herpetic esophagitis is self-limited and resolves in approximately seven days, rarely requiring treatment due to the persistence or severity of symptoms. On the other hand, symptoms tend to be more severe in immunocompromised patients, and complications such as hematemesis, perforation, and necrotizing esophagitis are more frequent, entailing worse outcomes [1,12]. As such, treatment with a 14-day course of acyclovir is imperative. Oral valacyclovir can be used alternatively, as can foscarnet in cases of resistance. Endoscopic findings may take up to two weeks to resolve [1,4].

The above-reported patient represents an extremely rare case of herpetic esophagitis with typical clinical, endoscopic, and histopathologic manifestations associated with intranasal corticosteroids in the absence of a typical immunosuppressive factor. ESRD and allergic rhinitis, although included in the patient’s history, are considered unlikely inciters. Various studies have demonstrated that although humoral immunity participates in HSV infection regulation, it is cellular immunity, with mechanisms involving CD4+, CD8+ T-cells, interferon-γ, and other cytokines, that plays the major role in HSV protection and elimination [13-15]. ESRD predominantly involves impairment of humoral and not cell-mediated immunity, rendering it an uncommon stressor for HE [16]. Moreover, despite the long course of the patient's renal disease, renal function was adequately regulated through hemodialysis, and no episodes of HE were noted in the past. Allergic rhinitis, on the other hand, despite being a chronic element of the patient's history, did not seem to elicit any previous episode of HSV esophagitis, which took place only after the recent administration of nasal corticosteroids. Furthermore, although long-term systemic corticosteroids and their lymphotoxic activity comprise a typical risk factor, cases of herpetic esophagitis due to non-systemic administration such as epidural or through inhalation have been reported, although rarely [17,18]. Finally, it is worth mentioning that the patient had no other cause of cellular immunocompromise, as he had not undergone transplantation, treatment with biological agents, or any recent course of systematic corticosteroids, and a laboratory examination for HIV was negative up to six months before the onset of symptoms. Lymphocytes were in the normal range before and at diagnosis and recent imaging was not indicative of any malignancy. In that manner, this may be the first reported case where prolonged intranasal corticosteroids might have acted as the main stressor leading to HSV esophagitis, with pre-existing GERD or allergic rhinitis functioning as possible predisposing factors [10]. Due to the persistence of symptoms for more than seven days, extensive ulceration as shown in endoscopy, and significant comorbidities, antiviral therapy was considered appropriate.

## Conclusions

Herpetic esophagitis is associated with considerable discomfort and is not exclusively limited to patients with immune suppression. Although morbidity and mortality correlate with the immunological status of the patient, this is not absolute and the immunocompetent can be severely affected as well, although less frequently. Additionally, the use of non-systemic corticosteroids may comprise an unusual but worth considering risk factor. Therefore, even in patients without overt cellular immunocompromise, symptoms, such as odynophagia, dysphagia, and epigastric pain, should not be ignored, and prompt work-up should be initiated, as HSV esophagitis can occur despite the absence of typical immunosuppressive factors.
